# DNA Methylation Patterns Provide Insights into the Epigenetic Regulation of Intersex Formation in the Chinese Mitten Crab (*Eriocheir sinensis*)

**DOI:** 10.3390/ijms26073224

**Published:** 2025-03-30

**Authors:** Shu-Jian Fang, Shu-Cheng Shao, Meng-Qi Ni, Ya-Nan Yang, Zhao-Xia Cui

**Affiliations:** 1 School of Marine Sciences, Ningbo University, Ningbo 315020, China; 2 Laboratory for Marine Biology and Biotechnology, Qingdao National Laboratory for Marine Science and Technology, Qingdao 266235, China

**Keywords:** *Eriocheir sinensis*, intersex, DNA methylation

## Abstract

DNA methylation is a form of epigenetic regulation that plays an important role in regulating gene expression of organisms. However, the DNA methylation pattern of intersex crabs has not yet been clarified. In order to reveal the DNA methylation in intersex *Eriocheir sinensis*, this study investigated the genome-wide DNA methylation profiles of female, male, and intersex individuals. The similar results across samples showed that the levels of cytosine methylation in the CG context were significantly higher than that in the CHG and CHH contexts. The methylation levels in the promoter region were higher than those in other functional element regions. We screened 149 differentially methylated genes (DMGs) in the promoter region between female and intersex crabs and 110 DMGs between male and intersex crabs. Three core gene networks were found in a comparison group of female and intersex crabs that involved heat shock proteins, ribosomes, and metabolism pathways; two core gene networks were found in the comparison group of male and intersex crabs that involved ribosomes and metabolism pathways. The six confirmed genes of *Hsc70*, *Hsp90*, *Rpl18*, *Acsl1*, *Yip2*, and *Rpl7* had lower methylation levels in the promoter region of intersex crabs than that of female and male crabs. However, six genes showed higher expression in intersex crabs than in female and male crabs. Our results reveal that DNA methylation is involved in the formation and maintenance of life activities of intersex crabs through the regulation of gene expression, enriching the DNA methylation library of the whole genome of *E. sinensis* and providing new insights for a better understanding of the epigenetic regulation of the formation of intersex *E. sinensis.*

## 1. Introduction

Epigenetic mechanisms are cellular processes that do not change DNA sequences but regulate gene expression [1]. DNA methylation modification is the most common epigenetic phenomenon and has become one of the most studied hotspots in the field of epigenetic research in recent years. Whole genome methylation sequencing is a high-definition sequencing technology that detects the methylation status of cytosine bases. Studies have shown that DNA methylation has a very important role in aquatic animal growth, development, sex reversal, and environmental adaptation. In aquatic animals, a large number of genome-wide methylation studies have been carried out, including on *E. sinensis* [2], *Daphnia magna* [3], Pacific oysters (*Crassostrea gigas*) [4], *Takifugu rubripes* [5], and *Monopterus albus* [6].

Intersex is a biological phenomenon characterized by the simultaneous presence of male and female traits [7]. Intersex may occur for a variety of reasons, including environmental contamination [8], parasitism [9], and genetic abnormalities [10]. These factors may disrupt the development and function of the gonads, as well as alter the homeostasis of sex hormones. In aquatic animals, including amphibians [11], fish [12], and crustaceans [13,14,15], the phenomenon of intersex has been reported. In the half-smooth tongue sole (*Cynoglossus semilaevis*), temperature affects DNA methylation and regulates sex determination-related genes, leading to masculinization in fish with a female genotype [16]. Environmental stimuli have been found to increase the methylation levels of the aromatase gene in bluehead wrasse (*Thalassoma bifasciatum*), inhibiting the maintenance of female gonads and ultimately causing sex reversal [17]. Besides DNA methylation modifications, histone demethylation can also trigger sex reversal in temperature-dependent sex determination in red-eared slider turtles (*Trachemys scripta elegans*) [18]. Thus, epigenetic regulatory mechanisms play a crucial role in sexual plasticity.

*E. sinensis* is regarded as an important aquatic resource due to its exceptional economic and ecological value. The occurrence of intersex individuals within this species may lead to significant economic losses in the aquaculture industry. However, DNA methylation in the formation of intersex individuals is still poorly understood. We have conducted research on the morphology, histology, sex genes, and transcriptomics of intersex *E. sinensis* [19,20,21]. In this study, we employed EM-seq technology to systematically analyze the genome-wide DNA methylation profiles of intersex crabs and normal crabs (female and male), thereby expanding the bioinformatics resources of the DNA methylation landscape in *E. sinensis*. Considering the biological specificity of intersex, studying DNA methylation in intersex crabs could offer new insights into its formation mechanisms.

## 2. Results

### 2.1. Sequencing Data Quality Control and Reference Genome Alignment

To assess the DNA methylation profiles in intersex (cm) and normal crabs (fm and mm), we conducted EM-seq. After quality control and sequencing data processing, valid data for all samples were obtained. The Q20 (%) and Q30 (%) were similar across samples. The content of guanine + cytosine (GC) ranged from 21.93% to 22.31% and did not differ significantly for cm, fm, and mm samples. The raw datum for cm was 49.51 Gb, which was lower than that of fm (57.8 Gb) and mm (54.8 Gb). After eliminating the low-quality sequencing data, 38.65 Gb of valid data remained for cm, which was also lower than that of fm (46.28 Gb) and mm (44.02 Gb) (Table 1). After enzyme treatment, the enzyme conversion of cm was 98.35%, which was between fm and mm. After mapping these data to the reference genome, the alignment ratio of cm was 43.33%, which was lower than fm and mm. The average methylation rate of C sites in cm was 3.5%, which was lower than fm (4.5%), and higher than mm (2.6%) (Table 2). The above results indicated that our data were up to standard and can be used for subsequent studies.

### 2.2. Genome-Wide DNA Methylation Profiles

**Figure 1:**
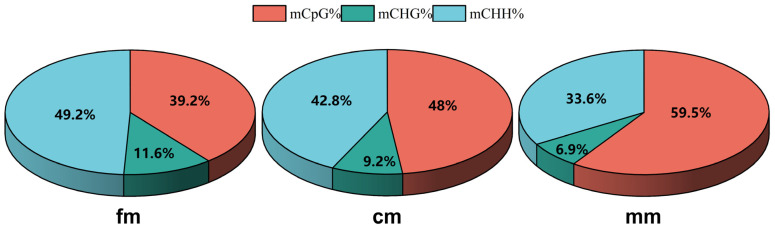
Overall methylation levels for fm, cm, and mm. The proportions of mCG, mCHG, and mCHH in mC. The red, blue, and green colors represent methylated (m) CG/mC, mCHH/mC, and mCHG/mC, respectively. mC represents cytosine methylation. H can be any of A, T, or C. fm, female crabs. mm, male crabs. cm, intersex crabs.

**Figure 2:**
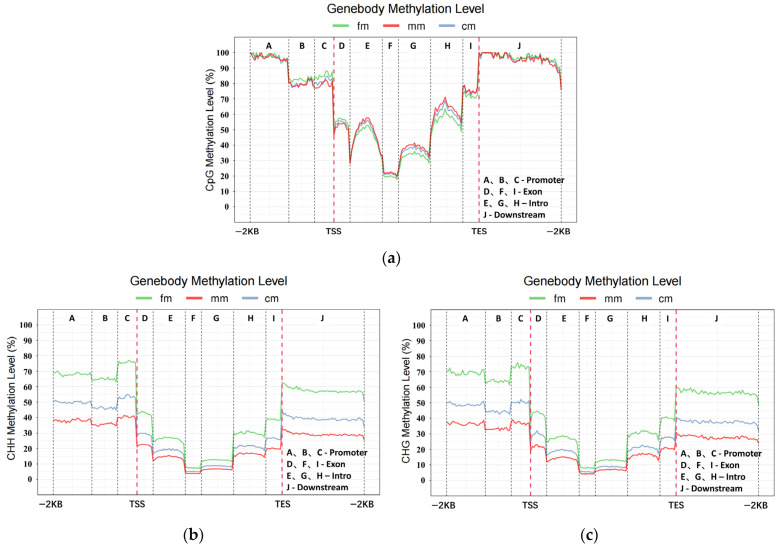
DNA methylation levels of three methylated forms of CG, CHH, and CHG among genomic functional elements. (**a**) CG, (**b**) CHH, and (**c**) CHG. H can be any of A, T, or C. The horizontal coordinates represent the different functional elements on the genome: A, B, and C are promoters. D, F, and I are exons. E, G, and H represent introns. J represents downstream. The left vertical coordinates indicate their methylation levels in their respective backgrounds. TSS is the transcription start site. TES is the transcription end site. fm, female crabs. mm, male crabs. cm, intersex crabs.

The cytosine methylation types were classified as CG, CHH, and CHG. H can be any of A, T or C. In intersex crabs, the average methylation rates of CG, CHH, and CHG were 48%, 42.8%, and 9.2%, respectively. In female crabs, the average methylation rates of CG, CHH, and CHG were 39.2%, 49.2%, and 11.6%, respectively. In male crabs, the average methylation rates of CG, CHH, and CHG were 59.5%, 33.6%, and 6.9%, respectively (Figure 1). The C-base methylation levels of different functional elements in the CG, CHG, and CHH sequences were analyzed. The functional elements (from 2 kb upstream of the transcription start site to 2 kb downstream of the transcription termination site) were categorized into promoter, exon, intron, and downstream regions (intergenic region) (Figure 2). The results showed that methylation in the intersex *E. sinensis* mainly happened in the CG sequence context, which was lower than that in males but higher than in females. However, intersex crabs were lower than that of females but higher than males for CHG and CHH. We found that the methylation levels in the promoter region were higher than those in other functional element regions.

We counted the number of DMRs in the cm vs. fm and cm vs. mm comparison groups. In the analysis of the cm vs. fm group, we identified 394,511 hyper-methylated DMRs and 3,393,915 hypo-methylated DMRs. In the analysis of the cm vs. mm group, we identified 2,553,753 hyper-methylated DMRs and 436,082 hypo-methylated DMRs (Table S1). It also showed that the methylation levels of intersex crabs were lower than those of female crabs and higher than those of male crabs.

### 2.3. Functional Enrichment of Promoter-Region DMGs

**Figure 3:**
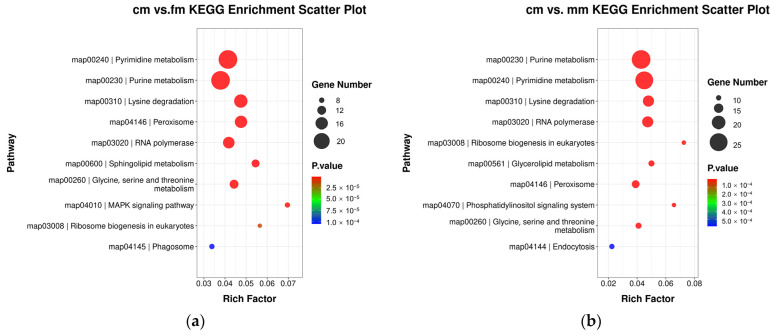
Kyoto encyclopedia of genes and genomes (KEGG) analysis of differentially methylated genes (DMGs) in the promoter region of cm vs. fm and cm vs. mm. (**a**) Scatter plot of KEGG pathways in the top 10 of cm vs. fm. (**b**) KEGG pathways for the top 10 of cm vs. mm. The ordinate represents the enriched pathway and the abscissa represents the Rich factor of the corresponding pathway. The size of the spots represents the number of DMGs enriched in each pathway, and the color of the spots represents the corrected *p*-value of each pathway. The Rich factor represents the ratio of the number of DMGs mapped to a pathway to the total number of genes mapped to that pathway. The larger the enrichment factor, the higher the degree of enrichment. fm, female crabs. mm, male crabs. cm, intersex crabs.

DMGs obtained from cm vs. fm and cm vs. mm groups were analyzed by GO (Figure S1) and KEGG enrichment. In both comparison groups, KEGG enrichment of the top 10 pathways in terms of number of genes was shown. Metabolism pathways, peroxisomes, RNA polymerases, and ribosome biogenesis in eukaryotes were common to the cm vs. fm and cm vs. mm groups. The MAPK signaling pathway and phagosomes were unique to the cm vs. fm group. Endocytosis and phosphatidylinositol signaling systems were unique to the cm vs. mm group (Figure 3).

### 2.4. PPI Networks of DMGs in the Promoter Region of Intersex and Normal Crabs

**Figure 4:**
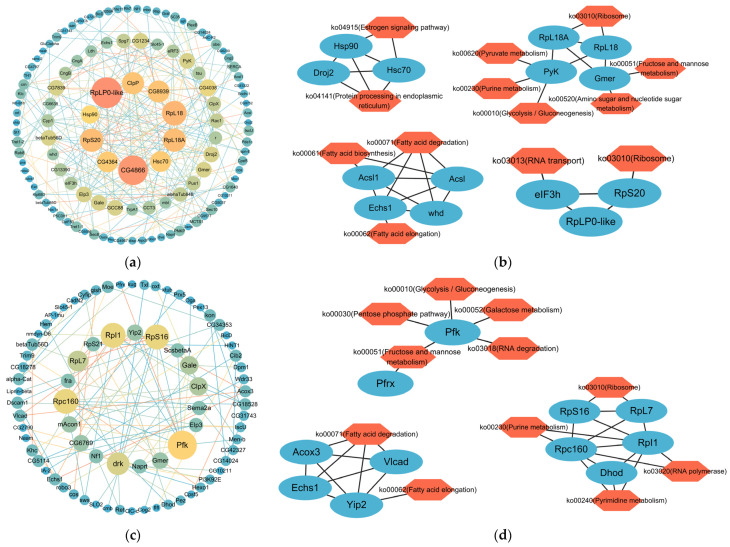
Protein–protein interaction (PPI) network and KEGG enrichment information for the cm vs. fm and cm vs. mm comparison groups. (**a**) The cm vs. fm PPI network of DMGs in the promoter region. (**b**) The core network of cm vs. fm identification using the MCODE plugin and enrichment analysis via the KEGG. (**c**) The cm vs. mm PPI network of DMGs in the promoter region. (**d**) The core network of cm vs. mm identification using the MCODE plugin and enrichment analysis via the KEGG. fm, female crabs. mm, male crabs. cm, intersex crabs.

In cm vs. fm, 460 DMGs in the promoter region were identified and enriched in specific pathways (Table S2). In the cm vs. mm group, 378 DMGs in the promoter region were found and enriched to specific pathways (Table S3). A PPI network containing 149 nodes (each representing a gene) and 279 edges (interactions between nodes) was constructed by analyzing 460 DMGs in the cm vs. fm group (Figure 4a). By using MCODE to analyze 149 genes, four key clusters of gene networks were identified, which were mainly associated with heat shock proteins, metabolism (fatty acid synthesis/degradation, purine metabolism, glycolysis/gluconeogenesis, fructose/mannose metabolism, amino acid/nucleotide metabolism and pyruvate metabolism), and the ribosomes (Figure 4b). The 378 DMGs screened in the cm vs. mm group were analyzed to form a PPI network containing 110 nodes and 130 edges (Figure 4c). Three key gene network clusters were screened, which were mainly involved in metabolism (fatty acid elongation/degradation, purine metabolism, glycolysis/gluconeogenesis, fructose/mannose metabolism, galactose metabolism, and pentose phosphate metabolism) and the ribosomes (Figure 4d). Among the metabolic pathways enriched, we found fatty acid elongation/degradation, purine metabolism, glycolysis/gluconeogenesis, and fructose/mannose metabolism were common to the cm vs. fm and cm vs. mm groups. However, amino acid/nucleotide metabolism and pyruvate metabolism were specific to the cm vs. fm group while galactose metabolism and pentose phosphate metabolism were specific to the cm vs. mm group.

### 2.5. Regulation of Gene Expression by Differential Methylation of Promoter Region

**Figure 5:**
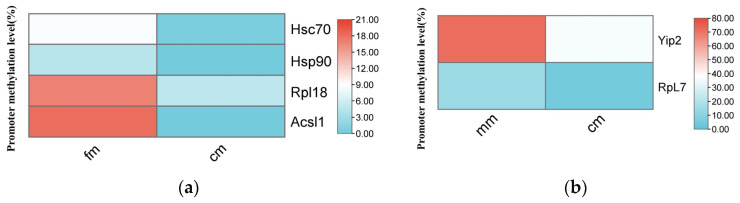
Methylation levels of DMGs in the promoter region. (**a**) Methylation levels of fm and cm key functional DMGs. (**b**) Methylation levels of mm and cm key functional DMGs. *Hsc70*, heat shock cognate protein 71 kDa. *Hsp90*, heat shock protein 90. *Rpl18*, 60S ribosomal protein L18. *Acsl1*, long-chain acyl-CoA synthetase 1. *Rpl7*, 60S ribosomal protein L7. *Yip2*, Yippee-interacting protein 2. fm, female crabs. mm, male crabs. cm, intersex crabs.

**Figure 6:**
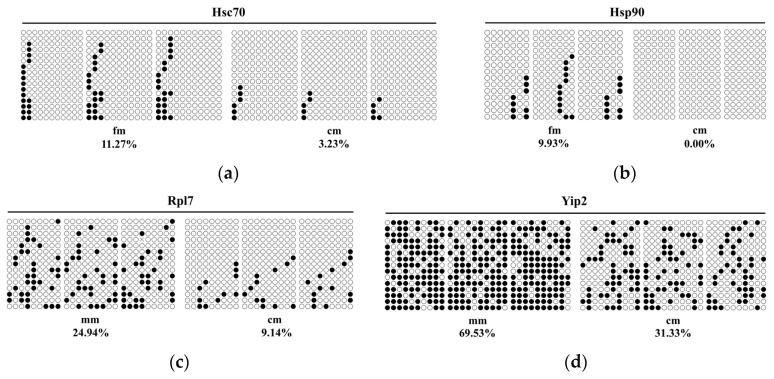
Validation of enzymatic methyl sequencing (EM-seq) data by bisulfite sequencing PCR (BSP). (**a**) BSP results of *Hsc70* gene between fm and cm. (**b**) BSP results of *Hsp90* gene between fm and cm. (**c**) BSP results of *Rpl7* gene between mm and cm. (**d**) BSP results of *Yip2* gene between mm and cm. Black and white circles indicate methylated and unmethylated CG, respectively. Cross indicates mismatch or gap in alignment. fm, female crabs. mm, male crabs. cm, intersex crabs.

**Figure 7:**
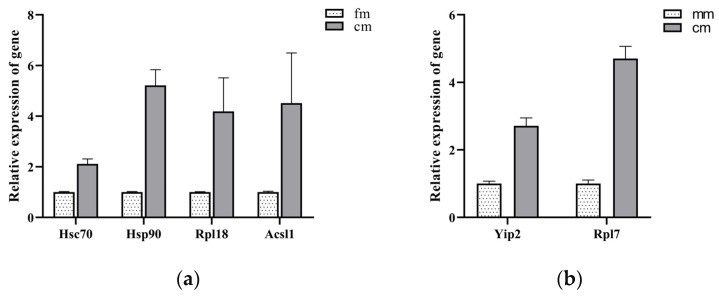
The relative expression of mRNA for the candidate gene in the muscle tissue of *E. sinensis* was detected using the qRT-PCR. (**a**) qRT-PCR of fm and cm key function DMGs (**b**) qRT-PCR of mm and cm key function DMGs. *18S* was used as an internal control. The values are represented as the mean ± SEM of three replicates. *Hsc70*, heat shock cognate protein 71 kDa. *Hsp90*, heat shock protein 90. *Rpl18*, 60S ribosomal protein L18. *Acsl1*, long-chain acyl-CoA synthetase 1. *Rpl7*, 60S ribosomal protein L7. *Yip2*, Yippee-interacting protein 2. fm, female crabs. mm, male crabs. cm, intersex crabs.

To investigate the impact of DNA methylation on the expression levels of key genes, we selected six key genes from the identified networks for validation. In the fm vs. cm group, heat shock cognate protein 71 kDa (*Hsc70*), heat shock protein 90 (*Hsp90*), 60S ribosomal protein L18 (*Rpl18*), and long-chain acyl-CoA synthetase 1 (*Acsl1*) were selected. In the mm vs. cm group, 60S ribosomal protein L7 (*Rpl7*) and Yippee-interacting protein 2 (*Yip2*) were selected. EM-seq data indicated that the methylation levels of the promoter region of these key genes were consistently higher in normal crabs compared with intersex crabs (Figure 5). The methylation levels obtained from BSP and EM-seq displayed a similar trend (Figure 6). Additionally, we employed qRT-PCR to assess the expression profiles of the selected key DMGs (Figure 7). The results demonstrated that the expression levels of these key DMGs were significantly upregulated in intersex crabs.

## 3. Discussion

DNA methylation is a form of epigenetic regulation with important roles in gene expression and tissue development [22]. As is known, arthropods exhibit low global genomic cytosine methylation levels, with some variability existing between crustaceans and insects (0–1% of total cytosines) [23]. However, the methylation levels of swimming crab (*Portunus trituberculatus*) range from 1 to 1.5% [24]. In this study, the genome-wide methylation levels of *E. sinensis* ranging from 2.6% to 4.5% were even higher than those of the swimming crab. DNA methylation levels in vertebrates are 5–10% [25]. The genome-wide methylation levels of *E. sinensis* were lower than those of vertebrates. Studies have shown that differences in the ability of species to adapt to their environments and the levels of evolution of species are important causes of differences in DNA methylation levels between species [26].

To our knowledge, this study represents the first comprehensive comparison of genome-wide DNA methylation patterns between normal and intersex crabs. There are differences among the three forms of cytosine methylation, with mCG being the predominant methylation type, which is consistent with the previous studies in other species [27,28,29,30]. By counting the number of DMRs and genome-wide methylation levels, we found that females are more methylated than intersex, and intersex are more methylated than males. The differences in DNA methylation levels between samples suggest that the intersex crab is an intermediate state between female and male crabs, which also corresponds to the sex marker identification as female. This difference may be the result of environmental stress and plays an important role in the formation of intersex crabs [31].

Heat shock proteins (HSPs) are a class of functionally similar proteins that alter gene expression in response to environmental stresses [32,33]. This family of genes is involved in the regulation of sex determination in several temperature-dependent sex-determining species [34,35,36]. After heat treatment, *Hsc70* overexpression in seminiferous cells of half-smooth tongue sole can affect the expression of the sex-related genes *sox9a* and *cyp19a1a*. *Hsc70* acts as a key regulator linking external high-temperature signaling to in vivo sex differentiation [37]. Similarly, *Hsc70* is a hub gene for pseudomale half-smooth tongue sole formation [38]. *Hsc70* and *Hsp90* can interact with steroid hormone receptors [39,40]. In our study, promoter regions of *Hsc70* and *Hsp90* show hypermethylation in female and hypomethylation in intersex crabs. Their expression levels is an opposite trend. These findings suggest that *Hsc70* and *Hsp90* genes may be regulated by DNA methylation. DNA methylation modifications of *Hsc70* and *Hsp90* may be involved in the changes in female crabs into intersex crabs by regulating the expression of these genes in response to environmental factors.

KEGG enrichment and PPI network analysis revealed that many DMGs were enriched in the ribosomal pathway between the normal and intersex crabs. A number of ribosomal protein genes as key candidate genes were identified. Studies have shown that ribosomal proteins can be used as candidate target genes for various environmental stresses (heavy metals, temperature) in aquaculture [41,42,43]. For instance, *Rpl7* promotes the proliferation of myoblasts and inhibits their differentiation by affecting ribosomal translation [44]. During the embryonic development of *Chironomus riparius*, the expression levels of *Rpl11* and *Rpl13* significantly increase, indicating the crucial role of ribosomal proteins in development [45]. In our study, we identified seven differentially methylated ribosomal protein genes (*RplP0*, *Rpl1*, *Rpl7*, *Rps16*, *Rpl18*, *Rpl18A,* and *Rps20*) in intersex crabs compared to normal crabs. *Rpl7* and *Rpl18* are hypomethylated in intersex crabs compared to normal crabs, and their gene expression levels are higher in intersex crabs. These findings suggest that these genes may have important significance in the formation of intersex crabs. The exact mechanisms remain unclear and warrant further investigation. Ribosomal proteins play a crucial biological role in regulating ribosomal structure and protein synthesis to maintain cellular homeostasis, and processes such as replication, transcription, RNA synthesis, and DNA repair [46,47]. The upregulation of these ribosomal genes may be due to increased ribosome synthesis rates required to support the normal growth and development of intersex crabs [48].

Many DMGs in the PPI network were also enriched in metabolic pathways. Among the metabolic pathways enriched in the cm vs. fm and cm vs. mm groups, both common and distinct pathways are present, but all of these pathways are relevant to energy metabolism. This implies that the energy metabolism of intersex crabs is active and higher than that of normal crabs. Some studies have shown that crustaceans increase their energy metabolism in response to various stressors [49,50,51]. Among these DMGs, *Acsl1* and *Yip2* were verified, and promoter region of *Acsl1* and *Yip2* genes showed hypermethylation in normal crabs and hypomethylation in intersex crabs. Their expression levels showed an opposite trend. The *Acsl1* gene encodes acyl-CoA synthetase, which functions in the conversion of free long-chain fatty acids into fatty acyl-CoA esters. It participates in the heat stress response by regulating fatty acid metabolism, which has been demonstrated in fish [52,53]. *Yip2* is a homolog of mammalian acetyl-CoA acyltransferase 2 (*ACAA2*), which catalyzes mitochondrial fatty acid β-oxidation [54]. This suggests that the intersex crabs may be in a state of stress, and the above pathways are adapted to this state of stress by providing energy to the intersex crabs.

## 4. Materials and Methods

### 4.1. Ethics Statement

Animal handling and experimental procedures were performed according to the Guide for the Use of Experimental Animals of Ningbo University. The study protocol was approved by the Animal Ethics Committee of Ningbo University.

### 4.2. Sample Collection and Classification

Intersex individuals (cm) of *E. sinensis* used in this study were collected from Xinghua, Jiangsu province, China. All samples had part of the sexual characteristics of a female or male. The external sex characteristics of these samples included a penis, pleopods, gonopods, abdominal appendage, gonopores, and ostioles of unknown function in the abdomen. These intersex crabs were previously identified as genetically female by our laboratory’s sex marker [19]. Normal mature female (fm) and male (mm) crabs were selected as control. Three crabs were selected for each sample. The muscle tissues from the three crabs were pooled into one test tube and rapidly frozen in liquid nitrogen until further use.

### 4.3. Total Genomic DNA Extraction

Total genomic DNA (gDNA) was extracted from muscle tissue. Total DNA was extracted using QIAamp Fast DNA Tissue Kit (Qiagen, Dusseldorf, Germany) following the manufacturer’s procedure. The quantity of DNA was measured by spectrophotometer. When the A260/280 ratio is between 1.8 and 2.0, DNA is available.

### 4.4. Enzymatic Methyl Sequencing (EM-seq)

The gDNA was acoustically sheared to an average size of 200–280 bp (peak approximately 250 bp) using the Covaris instrument. Two internal controls were added to each sample: unmethylated lambda and methylated pUC19 CG sites. Tet methylcytosine dioxygenase 2 (TET2) and T4-BGT enzymes were used to protect 5 mC and 5 hmC from deamination. Subsequently, APOBEC3A converted cytosines (but not protected forms of cytosines) into uracil. The converted DNA was then prepared into single-stranded DNA libraries using the Accel-NGS DNA Library Kit (Swift Biosciences, Ann Arbor, MI, USA) according to the manufacturer’s protocol. Briefly, truncated adapter 1 was ligated to the 3′ end of the single-stranded DNA. A new DNA strand was generated through an extension step, followed by the addition of truncated adapter 2 to the 5′ end of the DNA. Finally, PCR amplification was performed using full-length adapter 1 and adapter 2 to increase the yield of DNA molecules. The final libraries were sequenced on the NovaSeq 6000 platform at LC Sciences (Houston, TX, USA), using paired-end 2 × 150 bp sequencing.

### 4.5. Identification of Differentially Methylated Regions (DMRs)

DMRs were analyzed separately for cm vs. fm and cm vs. mm. Firstly, adapter contamination, low-quality bases, and undetermined bases were removed from the reads using Cutadapt [55] and Perl scripts. Sequence quality was then validated with FastQC (v0.11.9). Reads that passed quality control were mapped to the reference genome using Bismark [56]. Post-alignment, duplicate reads were further removed using Samtools [57]. DNA methylation levels at each cytosine site were calculated as the C/(C + T) ratio (methylated reads divided by total reads) using the software of custom scripts and MethPipe (v3.4.3) [58]. DMRs were calculated using the R package MethylKit (v1.26.0) [59] (sliding window 1000 bp, overlap 500 bp, *p*-value < 0.05).

### 4.6. Functional Enrichment Analysis of Differentially Methylated Genes (DMGs) in Promoter Region

The promoter region of genes are methylated, resulting in changes in gene expression levels. We performed functional enrichment analysis of genes that were differentially methylated in the overall promoter region (2500 bp upstream of the transcription start site). All DMGs in the following text refer to genes that are differentially methylated in the overall promoter region. Gene Ontology (GO) enrichment analysis for DMGs was conducted using the GOseq R package [60], with a *p*-value threshold of <0.05. Kyoto Encyclopedia of Genes and Genomes (KEGG) pathway enrichment of DMGs was assessed using KOBAS software (v3.0) [61].

### 4.7. PPI Analysis of DMGs in Promoter Region

We separately counted genes in the cm vs. fm and cm vs. mm comparison groups that had differential methylation levels in the promoter region and were enriched for specific GO and KEGG pathways. After obtaining the relevant genetic information, we conducted PPI analysis of DMGs in the promoter region obtained in comparison groups of cm vs. fm and cm vs. mm. The STRING database (v12.0) was employed for the construction of the PPI of proteins encoded by the intersection of DMGs. STRING is a database of known and predicted PPIs. The interactions include direct (physical) and indirect (functional) associations. The interactions were procured from genomic context, high-throughput experiments, co-expression, and previous knowledge. For a representative choice of database, either “*Drosophila*” or “*Mus musculus*” was selected, as no species-specific protein database is available for *E. sinensis* in STRING. Networks were therefore built to be representative of the phylum Arthropoda (with *Drosophila* showing most homology protein hits to *E. sinensis*) and Mammalia (using *Mus musculus*), respectively. Because the Drosophila database has the largest number of protein sequences and a closer evolutionary relationship, we finally chose *Drosophila melanogaster* as the reference organization. Parameters applied in STRING were “basic settings” and “medium confidence”. Cytoscape (v3.10.0) was applied to construct the DMG interaction network using the degree value as a criterion. A graph of theoretical clustering algorithm, molecular complex detection (MCODE) was utilized to analyze densely connected region. MCODE is part of the Cytoscape software. Further screening of the subnetwork containing hub DMGs was performed by the MCODE plugin of Cytoscape and the results were regarded as key functional DMGs. Module identification criteria included a degree cutoff of 2, node score cutoff of 0.2, k-core of 2, and maximum depth of 100. Significant modules were identified with MCODE score > 4.

### 4.8. Bisulfite Sequencing PCR (BSP)

Bisulfite sequencing PCR was used to validate the DNA methylation levels of selected candidate genes. Genomic DNA was treated with sodium bisulfite using the EZ DNA Methylation Kit (Zymo Research, Los Angeles, CA, USA). Following this, PCR amplification of the bisulfite-converted gDNA was carried out using Zymo Taq™ DNA Polymerase (Zymo Research). Details of the BSP primers are provided in Table 3. The PCR products were purified using a gel extraction kit (Sangon, Shanghai, China), then ligated and cloned into the pMD19-T vector (Sangon). Fifteen random clones from each sample were selected for DNA sequencing. Methylation analysis was performed using quantitative tools for bisulfite sequencing data (QUMA; v1.1.16).

### 4.9. Quantitative Real-Time PCR Analysis

Quantitative reverse transcription polymerase chain reaction (qRT-PCR) was utilized to detect the expression profiles of key functional DMGs to further understand the possible molecular regulatory mechanisms. The qRT-PCR was performed using the 7500 Real-Time PCR system (Applied Biosystems, Foster City, CA, USA) with TB Green Premix Dimer Eraser (2×) (TaKaRa, Kyoto, Japan). The qRT-PCR was carried out in a total volume of 20 μL, containing 10 μL TB Green Premix Dimer Eraser (2×), 6.4 μL RNase-free water, 2 μL diluted cDNA, 0.5 μL forward/reverse primers (1 mM), and 0.4 μL ROX Reference Dye II. Each sample was run in triplicates. All primers used for the qRT-PCR are listed in Table 4. A standard curve was used to calculate the amplification efficiency and ensure the primer specificity of each primer pair. The relative expression levels of target genes were calculated by the 2−ΔΔCt method [62]. The *18S* rRNA gene was used as an internal reference.

## 5. Conclusions

In summary, we have systematically characterized the whole-genome DNA methylation patterns in female, male, and intersex *E. sinensis* for the first time, and identified many DMRs and DMGs. Specifically, we identified 149 key DMGs in the promoter region between the cm and fm groups, and 110 key DMGs between the cm and mm groups. The key DMGs associated with the ribosomal pathway and metabolic pathway were enriched in both the cm vs. fm and cm vs. mm groups. Interestingly, only heat shock protein-associated DMGs were enriched in cm vs. fm. Our study provides information on the DNA methylation differences among females, males, and intersex crabs, which enriches the genome-wide DNA methylation library of *E. sinensis*.

## Figures and Tables

**Table 1:** Sequencing data quality control results.

Sample	Raw Data		Valid Data		Valid%	Q20%	Q30%	GC%
	Read	Base	Read	Base				
fm	385,311,926	57.80 G	374,807,550	46.28 G	97.27	97.19	93.44	22.31
mm	365,349,640	54.80 G	351,897,242	44.02 G	96.32	97.09	93.21	21.93
cm	330,057,373	49.51 G	321,911,061	38.65 G	97.54	97.24	93.60	22.13

Note: fm, female crabs; mm, male crabs; cm, intersex crabs.

**Table 2:** Sequencing data mapping to the reference genome.

Sample	Total Reads	Mapped Reads	Mapping Rate (%)	EM Conversion Rate (%)	Total mC (%)
fm	374,807,550	171,443,461	45.74	97.13	4.5
mm	351,897,242	161,322,433	45.84	99.06	2.6
cm	321,911,061	139,530,777	43.33	98.35	3.5

Note: fm, female crabs; mm, male crabs; cm, intersex crabs. mC represents cytosine methylation.

**Table 3:** Bisulfite Sequencing PCR primers.

Gene	Nucleotide Sequence (From 5′ to 3′)
*Hsc70*	inner-F: GTTATTTAGAGAGATAAAGATYGAGAG
	inner-R: CATTCAAAAAAACAACAATTCT
	outer-F: TTTTGAGGTTGTTTTGTGTAGG
	outer-R: CCAAACACACACATTTAACAAAC
*Hsp90*	inner-F: TGTTTTTTAGTTTGTGGTTTGT
	inner-R: AAAAATTTCCTCAAAATCAAAT
	outer-F: AGAYGTTTTTATTTTTATTTTAGAGGGG
	outer-R: CCCTAAAAAAACAATAAACATCCAAAAA
*Rpl7*	F: ATTGAAGGTTTGTAAATTGGAGT
	R: TTATAAAAAAAAACACATCCCAA
*Yip2*	F: TAGTGTATATTTGAAGGTGTTGAA
	R: AATAATAATAACCCAAACAAACAC

Note: *Hsc70*, heat shock cognate protein 71 kDa. *Hsp90*, heat shock protein 90. *Rpl7*, 60S ribosomal protein L7. *Yip2*, Yippee-interacting protein 2.

**Table 4:** Quantitative real-time PCR primers.

Gene	Nucleotide Sequence (From 5′ to 3′)
*Hsc70*	F: CGAGACCAAGTCGTTCTACCC
	R: GCAGCACATTGAGACCAGAGA
*Hsp90*	F: TCGCAGTTCATTGGCTATCC
	R: CCTCAATCTTGGGCTTCTCAT
*Rpl18*	F: GAACCCAAGTCGCAGGATG
	R: CACCAGCCCAGAGAGTGAGAT
*Acsl1*	F: TCCGTAACCTTGTGGATGACTA
	R: TTCTCCTTATCACACCCCTTTC
*Rpl7*	F: GCTCCGTTATTGTCCCTGC
	R: GTAGCCCCAGGCAATGAAG
*Yip2*	F: CTGTCAGGAACATTCGCTTTG
	R: CTTCATTCTGGGCGGTAGC

Note: *Hsc70*, heat shock cognate protein 71 kDa. *Hsp90*, heat shock protein 90. *Rpl18*, 60S ribosomal protein L18. *Acsl1*, long-chain acyl-CoA synthetase 1. *Rpl7*, 60S ribosomal protein L7. *Yip2*, Yippee-interacting protein 2.

## Data Availability

The data used in this article have been submitted to the NCBI database. (SUB15104681).

## Supplementary Materials

The following supporting information can be downloaded at: https://www.mdpi.com/article/10.3390/ijms26073224/s1.
