# Supplementary figures and images for: DNA Methylation Patterns Provide Insights into the Epigenetic Regulation of Intersex Formation in the Chinese Mitten Crab (Eriocheir sinensis)
Source: Int J Mol Sci. 2025 Mar 30;26(7):3224. doi: 10.3390/ijms26073224 (PMC11989155; doi:10.3390/ijms26073224)

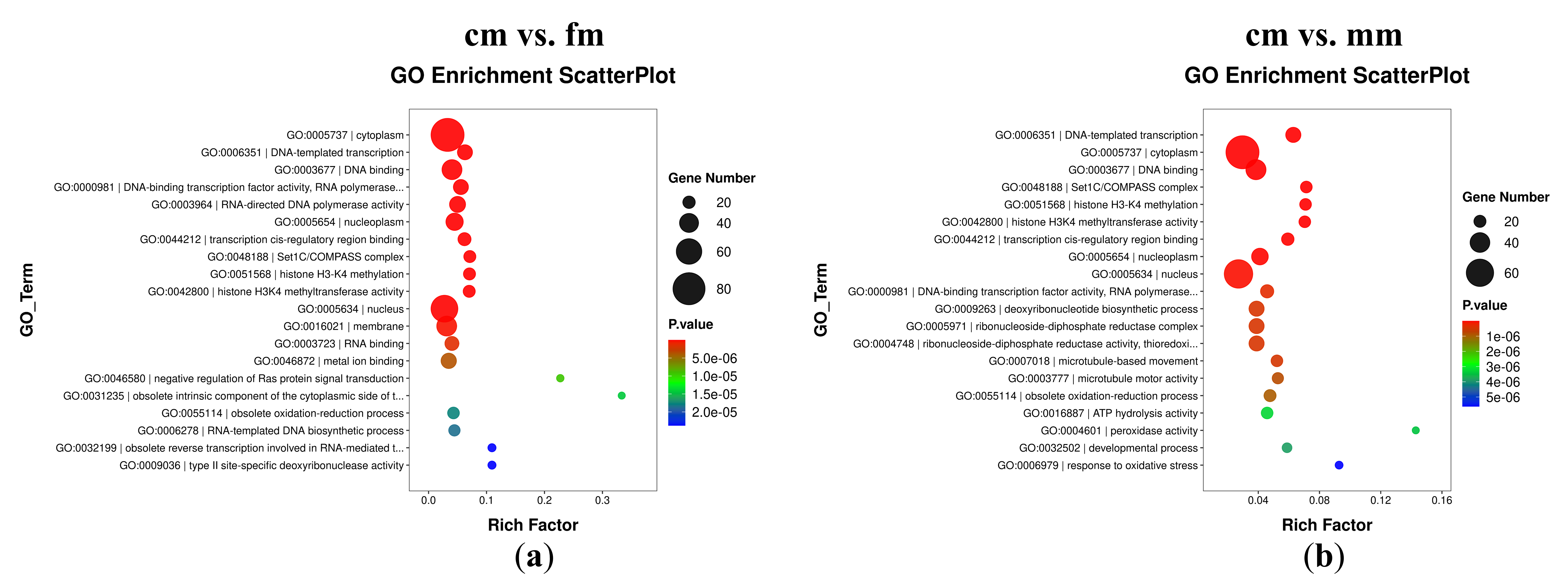

Supplement: Supplementary file 1 [file ijms-26-03224-s001.zip › Supplementary File/Figure S1 cm vs. fm and cm vs. mm groups promoter DMGs GO enrichment.tif]
